# ﻿Four new species of Dothideomycetes (Ascomycota) from Pará Rubber (*Heveabrasiliensis*) in Yunnan Province, China

**DOI:** 10.3897/mycokeys.103.117580

**Published:** 2024-03-22

**Authors:** Rui-Fang Xu, Samantha C. Karunarathna, Chayanard Phukhamsakda, Dong-Qin Dai, Abdallah M. Elgorban, Nakarin Suwannarach, Jaturong Kumla, Xiao-Yan Wang, Saowaluck Tibpromma

**Affiliations:** 1 Center for Yunnan Plateau Biological Resources Protection and Utilization, College of Biological Resource and Food Engineering, Qujing Normal University, Qujing, Yunnan 655011, China; 2 Center of Excellence in Fungal Research, Mae Fah Luang University, Chiang Rai, Thailand; 3 School of Science, Mae Fah Luang University, Chiang Rai, 57100, Thailand; 4 National Institute of Fundamental Studies (NIFS), Kandy, Sri Lanka; 5 Department of Botany and Microbiology, College of Science, King Saud University, Riyadh 11451, Saudi Arabia; 6 Department of Biology, Faculty of Science, Chiang Mai University, Chiang Mai, Thailand; 7 Center of Excellence in Microbial Diversity and Sustainable Utilization, Chiang Mai University, Chiang Mai, Thailand; 8 Edible Fungus Research Institute of Hunan Province, Changsha 410013, China; 9 Luliang cuan Lu yuan Mushroom Co., LTD, Luliang 655607, China

**Keywords:** Dothideomycetes, four new species, multigene phylogeny, Pará rubber, saprobic fungi, taxonomy

## Abstract

The tropical areas in southern and south-western Yunnan are rich in fungal diversity. Additionally, the diversity of seed flora in Yunnan Province is higher than in other regions in China and the abundant endemic species of woody plants provide favourable substrates for fungi. Rubber plantations in Yunnan Province are distributed over a large area, especially in Xishuangbanna. During a survey of rubber-associated fungi in Yunnan Province, China, dead rubber branches with fungal fruiting bodies were collected. Morphological characteristics and multigene phylogenetic analyses (ITS, LSU, SSU, *rpb*2 and *tef*1-α) revealed four distinct new species, described herein as *Melomastiapuerensis*, *Nigrogranalincangensis*, *Pseudochaetosphaeronemalincangensis* and *Pseudochaetosphaeronemaxishuangbannaensis*. Detailed descriptions, illustrations and phylogenetic trees are provided to show the taxonomic placements of these new species.

## ﻿Introduction

*Heveabrasiliensis* (Pará rubber tree) is native to the Amazon River Basin; however, it shows a pantropical species distribution through introductions (Basik et al. 2021). Pará rubber plantations have increased intensely worldwide in the past few decades, with the global consumption of natural rubber increasing by about 3% in 2019 (Bhattacharjee et al. 2021). Yunnan Province is one of the rubber-producing provinces in China and Xishuangbanna Prefecture (located in the south of Yunnan) contributes up to 77% of the rubber production in the province, representing 37% of the national rubber production (National Bureau of Statistics of China 2011, Statistical Bureau of Yunnan Province 2011).

Besides rubber, Yunnan Province is also rich in fungal diversity (Feng and Yang 2018). Approximately 104,000 fungal species are expected to be discovered in Yunnan; however, around 6,000 fungal species have been reported from the Province, leaving much to be described (Feng and Yang 2018; Bhunjun et al. 2022; Phukhamsakda et al. 2022). Surprisingly, a few fungal species have been described on Pará rubber in China (Senwanna et al. 2021; Xu et al. 2022a, 2022b, 2023; Hyde et al. 2023).

Senwanna et al. (2021) listed 67 orders, 168 families and 513 genera of fungi on Pará rubber and reported eight new taxa, two asexual-sexual linkages, 20 new host records and one reference specimen of saprobic fungi from Thailand. In addition, Senwanna et al. (2021) reported that three species from their collections had previously been reported from Pará rubber in the Amazon Forest (Spaulding 1961) and most of the taxa reported on Pará rubber have been found in Thailand. Moreover, Senwanna et al. (2019) discovered that *Muyocoprondipterocarpi* may have jumped from its original host. *Dipterocarpustuberculatus*, to the Pará rubber host and adapted to the new host in Thailand.

Dothideomycetes is the largest class of Ascomycota, currently encompassing more than 25 orders, 110 families and over 19,000 species (Wijayawardene et al. 2022). They can be endophytes, epiphytes, saprobes, lichenised or lichenicolous fungi and are found in terrestrial, freshwater and marine habitats worldwide (Hyde et al. 2013). In Pará rubber, Dothideomycetes are predominant amongst ascomycetes (Senwanna et al. 2021).

Fungi associated with rubber in China were poorly studied compared with other countries in the Greater Mekong Subregion (GMS), especially in Thailand. Moreover, saprobic fungal taxa, described in earlier studies, do not have sequence data. Continuing the fungal diversity studies in the GMS (Chaiwan et al. 2021), this study introduces four new taxa of Dothideomycetes associated with Pará rubber trees in Yunnan Province, China. Morphological characteristics and phylogenetic analyses were conducted to find accurate taxonomic placements of these new taxa.

## ﻿Materials and methods

### ﻿Collection, morphological examination and isolation

Dead rubber (*Heveabrasiliensis*) branches with fungal fruiting bodies were collected from Yunnan Province, China, during the summers of 2021 and 2022. The samples were stored in sealable plastic bags and taken to the mycology laboratory at Qujing Normal University. Morphological observations and single spore isolations were conducted following the methods described by Senanayake et al. (2020). Morphological characteristics were observed using a stereomicroscope Leica S8AP0 and photographed with an OLYMPUS BX53 compound microscope. Measurements were obtained using Tarosoft (R) Image Frame Work software. Adobe Photoshop CC 2017 software was used for preparing photo-plates. Herbarium specimens of the new species were deposited at the Herbarium of Zhongkai University of Agriculture and Engineering (ZHKU), China. The living cultures were deposited at the culture collection of Zhongkai University of Agriculture and Engineering (**ZHKUCC**), China. Facesoffungi (FoF) numbers and Index Fungorum (IF) numbers were obtained as per Jayasiri et al. (2015) and Index Fungorum (2024).

### ﻿DNA extraction, PCR amplification and sequencing

Genomic DNA was extracted directly from scraped fresh mycelia grown on one-month-old artificial culture media (PDA), using an E.Z.N.A. Forensic DNA Kit (BIO-TEK), in accordance with the manufacturer’s protocol. The different gene regions, primers and protocols used for the amplification are summarised in Table 1. Polymerase chain reaction (PCR) amplifications were conducted using 25 μl PCR mixture containing 8.5 μl ddH2O, 12.5 μl 2 × Master Mix (Bioteke Corporation, Beijing, China), 2 μl DNA template and 1 μl each reverse and forward primer (Tibpromma et al. 2018). Purification and sequencing of PCR products were carried out in Bioteke, P.R. China.

**Table 1.** Primers, PCR thermal cycles for SSU, ITS, LSU, *rpb*2 and *tef*1-α amplification and reference(s).

Genes	Primers/Loci	PCR condition	References
ITS	ITS4	(94 °C: 30 s, 55 °C: 50 s, 72 °C: 90 s) × 35 cycles	White et al. (1990)
	ITS5		
LSU	LR0R	(94 °C: 30 s, 55 °C: 50 s, 72 °C: 90 s) × 35 cycles	Vilgalys and Hester (1990)
	LR5		
SSU	NS1	(94 °C: 30 s, 55 °C: 50 s, 72 °C: 90 s) × 35 cycles	White et al. (1990)
	NS4		
*tef*1-α	983F	(95 °C: 30 s, 55 °C: 50 s, 72 °C: 90 s) × 35 cycles	Carbone and Kohn (1999)
	2218R		
*rpb*2	fRPB2-5f	(94 °C: 60 s, 58 °C: 60 s, 72 °C: 90 s) × 40 cycles	Liu et al. (1999)
	fRPB2-7cR		

### ﻿Phylogenetic analyses

Sequences with high similarities (> 90%) were identified by BLASTn searches to determine the closest match to the taxa. Initial alignments of the sequence data were processed using MAFFT v.7 (http://mafft.cbrc.jp/alignment/server) using default settings (Katoh et al. 2019). The sequences were trimmed using TrimAl V 1.2 with ‘gappyout’ automated trimming option (Capella-Gutiérrez et al. 2009). The alignments were checked visually and improved manually wherever necessary. Multiple genes were concatenated by Sequence Matrix.

**Figure 1.**
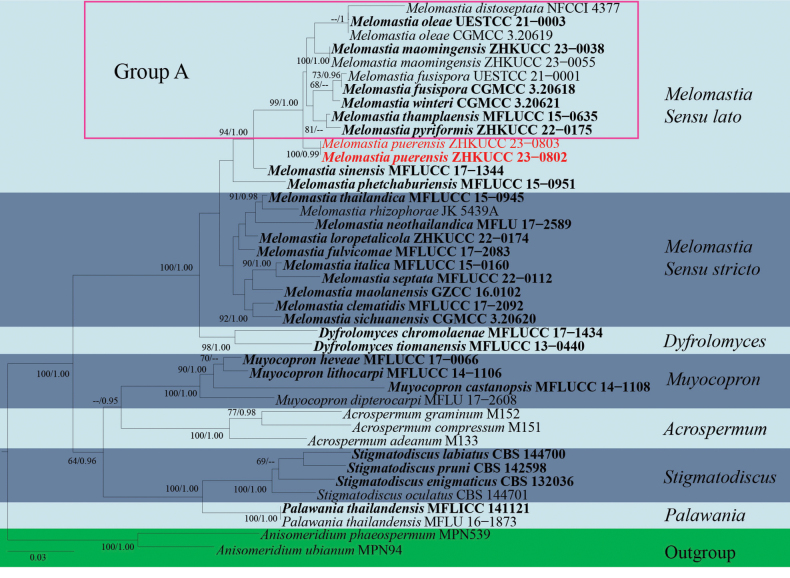
Phylogram generated from Maximum Likelihood analysis, based on combined LSU, SSU and *tef*1-α sequence data of 41 taxa, which comprised 2836 base pairs (LSU = 902 bp, SSU = 1031 bp, *tef*1-α = 903 bp). The best scoring RAxML tree with a final likelihood value of -14798.632437 is presented. The matrix had 1013 distinct alignment patterns, with 24.90% of undetermined characters or gaps. Estimated base frequencies were as follows: A = 0.241740, C = 0.258134, G = 0.292403, T = 0.207722; substitution rates: AC = 0.834723, AG = 2.021967, AT = 1.126143, CG = 1.032150, CT = 7.231944, GT = 1.000000; gamma distribution shape parameter α = 0.320795. Bootstrap support values for ML equal to or greater than 60% and Bayesian Inference analysis values equal to or greater than 0.90 PP are labelled at each node. The tree is rooted with *Anisomeridiumphaeospermum* (MPN539) and *A.ubianum* (MPN94). Related sequences were collected following Li et al. (2022), Kularathnage et al. (2023) and Dong et al. (2023). The new isolates are indicated in red and the ex-type strains are in bold. Group A indicates the taxa used to compare the morphology with our new species (*Melomastiapuerensis*).

Multigene phylogenetic analyses for the concatenated genes were conducted using Maximum Likelihood (ML) and Bayesian Inference (BI) analyses. The CIPRES Science Gateway portal (Miller et al. 2012) was used to run both RAxML and Bayesian analyses. Maximum Likelihood analysis was made with RAxML-HPC2 on XSEDE v.8.2.10 tool (Stamatakis 2014) employing the GTR+GAMMA model with 1000 bootstrap repetitions. Bayesian analysis was performed by MrBayes v.3.0b4 (Huelsenbeck and Ronquist 2001) with the best-fit model of sequence evolution estimated using MrModelTest 2.2 (Nylander 2004). MrBayes analyses were performed with GTR+I+GAMMA for one million generations, sampling every 100^th^ generation and ending the run automatically when the standard deviation of split frequencies dropped below 0.01 with a 25% burn-in. Phylograms were visualised with the FigTree v.1.4.0 programme (Rambaut 2012) and edited in Microsoft PowerPoint 2021. The final alignments and trees were deposited in TreeBASE, under submission ID 31039 (Fig. 1) and ID 31040 (Fig. 3) (http://www.treebase.org/).

## ﻿Results

### ﻿Taxonomy and phylogenetic results

**Dothideomycetes O.E. Erikss. & Winka**

**Dyfrolomycetales Pang, K.D. Hyde & E.B.G. Jones**

#### ﻿ Pleurotremataceae

Taxon classificationFungiDyfrolomycetalesPleurotremataceae

Watson

07931BC7-38F4-5875-960A-F07D4A52175C

##### Notes.

Pleurotremataceae was introduced by Watson (1929) and it comprises three genera viz. *Dyfrolomyces*, *Melomastia* and *Pleurotrema* (Wijayawardene et al. 2022). Species in this family are saprobes on wood in terrestrial and aquatic habitats (Hongsanan et al. 2020). Pleurotremataceae has been classified in several orders. Pleurotremataceae was excluded from Sordariomycetes and placed in Dothideomycetes, based on morphology and DNA sequences (Maharachchikumbura et al. 2016).

#### ﻿ Melomastia

Taxon classificationFungiDyfrolomycetalesPleurotremataceae

Nitschke ex Sacc.

8EFACC3C-4048-570A-815E-A64DB3C825D3

##### Notes.

*Melomastia* was introduced by Saccardo (1875) with *M.mastoidea* as the type species (Kang et al. 1999). *Melomastia* has been recorded with 63 epithets in Index Fungorum (2024). Most *Melomastia* species have been found in terrestrial, freshwater and marine habitats and they have a wide geographical distribution in Africa, China, Germany, Italy, Japan, Poland and the United States of America (Norphanphoun et al. 2017; Dayarathne et al. 2020; Li et al. 2022; Kularathnage et al. 2023). *Melomastia* was discovered to be closely related to *Dyfrolomyces* and their exact relationship is still unknown. Li et al. (2022) reclassified *Dyfrolomyces* as *Melomastia*, based on morphology and phylogeny of four newly-introduced species from Olive in Sichuan Province, China. *Melomastiatiomanensis* and *M.chromolaenae* exhibit spindle-shape, 6–11-septate ascospores with acute ends. Additionally, the phylogenetic analysis conducted by Kularathnage et al. (2023) showed that *M.tiomanensis* and *M.chromolaenae* form a distinct lineage. Thus, *M.tiomanensis* and *M.chromolaenae* were moved into *Dyfrolomyces* and named *Dyfrolomycestiomanensis* and *Dyfrolomyceschromolaenae*. *Melomastia* is characterised by immersed, ostiolate ascomata, multiple layered, dark brown peridium, filamentous pseudoparaphyses, unitunicate, cylindrical, 8-spored asci and ovoid, hyaline, 1–10-septate, fusiform to oblong ascospores with rounded or acute ends, with or without gelatinous sheath (Norphanphoun et al. 2017; Dayarathne et al. 2020; Li et al. 2022; Kularathnage et al. 2023). However, the asexual morph of *Melomastia* is still unknown (Norphanphoun et al. 2017; Li et al. 2022; Kularathnage et al. 2023).

#### ﻿ Melomastia puerensis

Taxon classificationFungiDyfrolomycetalesPleurotremataceae

R.F. Xu & Tibpromma sp. nov.

2A8B05E7-481A-522A-B739-D3BF341077D4

Index Fungorum number: IF901419

Facesoffungi number: FoF15195

Fig. 2

##### Etymology.

The name refers to the location “Pu’er, Yunnan, China”, where the holotype was collected.

##### Holotype.

ZHKU 23–0106.

##### Description.

***Saprobic*** on a dead branch of *Heveabrasiliensis*. ***Sexual morph***: Ascomata 260–720 μm high, 225–850 μm diam. (x‾ = 540 × 520 μm, n = 10), visible as black dots on the host surface, solitary or gregarious, immersed to slightly erumpent, subglobose or pyriform, carbonaceous, dark brown to black, ostiolate, papillate. Ostioles 205–220 × 195–258 µm (x‾ = 233 × 207 μm, n = 5), central, carbonaceous, dark brown to black. Peridium 40–120 µm wide, two-layered, outer layer, thick, carbonaceous, inner layer composed of several layers, brown to pale brown cells of textura angularis. Hamathecium comprises 2–4.5 μm wide, filiform, unbranched, hyaline, aseptate, guttulate, pseudoparaphyses, longer than asci. Asci 175–205 × 6–10 μm (x‾ = 190 × 8, n = 15), 8-spored, hyaline, bitunicate, cylindrical, flexuous, apically obtuse, with an ocular chamber, smooth-walled, short pedicellate. Ascospores 20–30 × 5–8 μm (x‾ = 24 × 7, n = 30), uniseriate, hyaline, fusiform, obtuse or conical ends, narrow towards the apex, 3-septate, constricted at the central septum, with guttulate in each cell. ***Asexual morph***: Undetermined.

**Figure 2.**
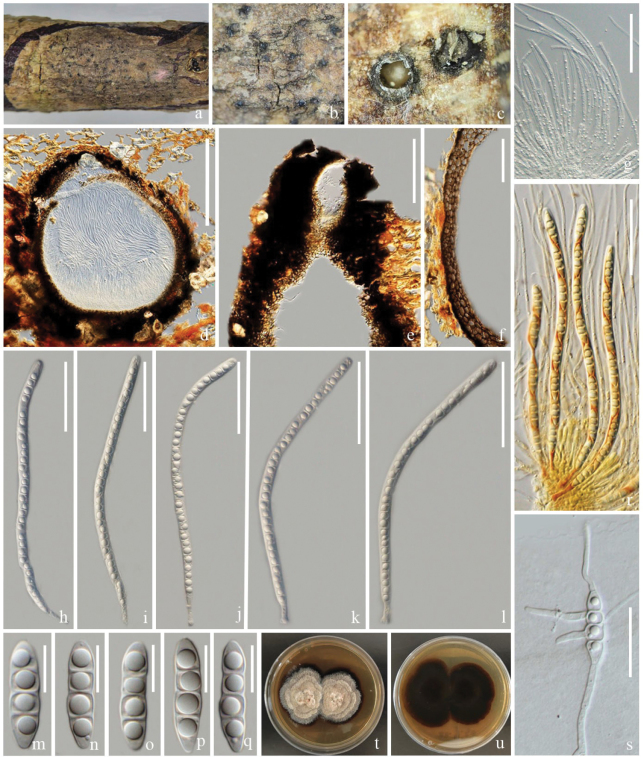
*Melomastiapuerensis* (ZHKU 23–0106, **holotype**) **a–c** appearance of ascomata on host surface **d** vertical section of an ascoma **e** vertical section of ostiole **f** section of peridium **g** hamathecium **h–l** asci **m–q** ascospores **r** asci stained in Lugol’s iodine **s** germinated ascospore **t, u** colonies on PDA (t-front and u-reverse views). Scale bars: 100 µm **(d–f)**; 50 µm **(g–l)**; 10 µm **(m–q)**; 30 µm **(s)**.

##### Culture characteristics.

Colonies on PDA that grow at 28 °C, flat, rough surface, entire edges, culture from above, brownish-grey, forming zonate grey, reverse dark brown, brown at the edge, turning reddish-brown.

##### Material examined.

China, Yunnan Province, Pu'er on a dead branch of *Heveabrasiliensis*, 16 September 2021, Rui-Fang Xu, XPER–14 (ZHKU 23–0106, holotype); ex-type ZHKUCC 23–0802, ZHKUCC 23–0803.

##### GenBank numbers.

ZHKUCC 23–0802 = ITS: OR941077, LSU: OR922309, SSU: OR922340, *tef*1-α: OR966284; ZHKUCC 23–0803 = ITS: OR941078, LSU: OR922310, SSU: OR922341, *tef*1-α: OR966285.

##### Notes.

The phylogenetic analyses showed that *Melomastiapuerensis* clustered basal to *M.distoseptata*, *M.fusispora*, *M.maomingensis*, *M.oleae*, *M.pyriformis*, *M.thamplaensis* and *M.winteri* with 99% MP, 1.00 PP support (Fig. 1). We compared the morphology of our collection with closely-related species and the differences are mentioned in Table 2. Our collection has slight differences from other closely-related species by having larger ascomata and wider peridium, but the phylogenetic tree shows that they are different species (Fig. 1, Table 2). Therefore, we introduce *M.puerensis* as a new species, based on morphology and phylogenetic analyses.

**Table 2.** Morphological comparison of *M.puerensis* and closely-related species viz. *M.distoseptata*, *M.fusispora*, *M.maomingensis*, *M.oleae*, *M.pyriformis*, *M.thamplaensis* and *M.winteri*.

Species	Ascomata	Peridium	Pseudoparaphyses	Asci	Ascospores	References
*M.distoseptata*	550–630 × 450–600 μm, perithecial, immersed, erumpent neck with pseudoparaphyses, clypeate	40 μm, with two strata, outer thick, and inner brown and hyaline cells of textura angularis to *epidermoidea* cells	1.8–2.1 μm, flamentous, septate, unbranched, dense, longer than asci	126.7–146.2 × 4.7–6.3 μm, apical ends obtuse, short pedicellate	19.7–24.9 × 4.3–5 μm, fusoid, obtuse ends, apical ends slightly bent	Hongsanan et al. (2020)
*M.fusispora*	432–624 × 527–618 μm, cone-shaped structures on the host surface, immersed to erumpent through host tissue, pyriform	25.5–61.5 µm, two-layered, outer layer of cells of *textura intricata*, inner layer of textura angularis	2–2.6 µm, dense, filiform, unbranched, hyaline, aseptate	200–231 × 7.6–9.2 µm, slightly flexuous, apically round, with well-developed ocular chamber, cylindrical pedicellate	27.5–32 × 6.5–7.5 µm, fusiform, with rounded to acute ends, narrow towards apex, constricted at the central septum, surrounded by an irregular and thin gelatinous sheath	Li et al. (2022)
*M.maomingensis*	300–550 µm high × 250–500 µm diam., solitary, semi-immersed to immersed, visible on the host surface as black, obvious, raised spots, black, uni-loculate, globose to subglobose	35–100 µm wide, comprising dense, thick, brown to dark brown cells of textura angularis, fusion with host tissue	1.5–3.5 µm wide, comprising numerous, filamentous, hyaline, septate, sometimes branched, longer than asci, attached at the base and between the asci	175–195 × 7–9 µm, cylindrical pedicel, rounded in apex, J-	(23–)24.5–29 × 6–8 µm, fusiform with acute ends, constricted at the septum, with a large guttule in each cell when mature	Du et al. (2024)
*M.oleae*	410–440 × 493–520 µm, cone-shaped structures on host surface, semi-immersed, globose to compressed globose	54–65 µm, two-layered, outer thick and inner composed of 5–6 layers of textura angularis to textura prismatica	2–2.5 µm, dense, filiform, unbranched, aseptate	209–237 × 7.5–9 µm, slightly flexuous, apically rounded with ocular chamber, cylindrical pedicellate	28–34 × 6–7 µm, fusiform with obtuse ends, slightly constricted at the septa	Li et al. (2022)
***M.puerensis***	**260–720 × 225–850 μm, black dot on the host surface, immersed to erumpent to superficial, pyriform**	**40–120 µm, two-layered, outer thick, carbonaceous, inner composed of several layers, pale brown to brown cells of *textura angularis***	**2–4.5 μm, filiform, unbranched, guttulate, pseudoparaphyses, longer than asci**	**175–205 × 6–10 μm, flexuous, apical ends obtuse, with ocular chamber, smooth-walled, short pedicellate**	**20–30 × 5–8 μm, fusiform, obtuse or conical ends, narrow towards apex, constricted at the central septum, with guttules in each cell**	**This study**
*M.pyriformis*	330–640 × 275–420 μm, erumpent to superficial when mature, pyriform, papillate, ostiolate	20–50 μm, thin at the base and become thick towards sides, comprised of brown, thick-walled, cells of *textura intricata* in sides; and thin-walled, pale brown, cells of textura angularis in base	1.8–2.5 μm wide, dense, filiform, unbranched, septate, anastomosing between and above the asci	135–160 × 5.5–7.5 μm, fissitunicate, apically round, with an indistinct ocular chamber, short pedicellate	20–25 × 4.5–7 μm, , fusiform with acute ends, not constricted at the septa, with guttules in each cell	Kularathnage et al. (2023)
*M.thamplaensis*	550–630 × 450–600 μm, black spots on the host surface, immersed, clypeate, subglobose to obpyriform, some with a broad, flattened base	14–49 μm, composed of three strata, an outer stratum, dense, amorphous, thick-walled cells fusing with host tissue, a middle layer of thick-walled, black cells of textura angularis and an inner layer of thin-walled black cells of textura angularis	1.8–2.1 μm, attached at the base and between the asci, embedded in a gelatinous matrix	126.7–146.2 × 4.7–6.3 µm, long cylindrical, short-pedicellate, apically rounded with an obvious apical ring	19.7–24.9 × 4.3–5 μm, fusiform with acute angular ends, constricted at the septum, smooth-walled, containing several guttules when young	Zhang et al. (2017)
*M.winteri*	340–365 × 364–410 µm, semi-immersed to immersed, globose	55–62.5 µm, two-layered, outer thick, and inner composed of 3–4 layers of hyaline to lightly brown cells of textura angularis to textura prismatica	1.5–3.5 µm, dense, filiform, unbranched, septate	165–189 × 7–8.5 µm, slightly flexuous, apically round, with a distinct ocular chamber, cylindrical pedicellate	25–30 × 5–6.5 µm, partially overlapping, fusiform with acute ends, deeply constricted at the median septum	Li et al. (2022)

###### ﻿Pleosporales Luttrell ex M.E. Barr

#### ﻿ Nigrogranaceae

Taxon classificationFungiDyfrolomycetalesPleurotremataceae

Jaklitsch & Voglmayr

9E017A5B-4E34-573E-8EE4-07D8DE94A085

##### Notes.

Nigrogranaceae was introduced by Jaklitsch and Voglmayr (2016), with *Nigrograna* as the type genus. The members of Nigrogranaceae can be found on a wide range of hosts in marine and terrestrial habitats (Dayarathne et al. 2020; Boonmee et al. 2021; Lu et al. 2022; Hyde et al. 2023).

#### ﻿ Nigrograna

Taxon classificationFungiDyfrolomycetalesPleurotremataceae

Gruyter, Verkley & Crous

7C008AE2-4F41-5071-8E64-AB8BD08F2D46

##### Notes.

*Nigrograna* was introduced by De Gruyter et al. (2013) with *N.mackinnonii* as the type species. *Nigrograna* has 32 epithets in Index Fungorum (2024). Ahmed et al. (2014) transferred *N.mackinnonii* to *Biatriospora*, based on multigene phylogenetic analysis. Kolařík et al. (2017) introduced four new endophytic species viz. *B.antibiotica*, *B.carollii*, *B.peruviensi*, and *B.yasuniana* in *Biatriospora*, based on morphology and multigene phylogeny and, later, Kolařík (2018) synonymised these four species under *Nigrograna*. The sexual morph of *Nigrograna* is characterised by globose, immersed or less commonly superficial ascomata, bitunicate, fissitunicate 8-spored asci with short stipe and knob-like base, asymmetric, fusoid, 1–3-septate, pale to chocolate brown, smooth or faintly verrucose ascospores (Jaklitsch and Voglmayr 2016). The asexual morph is characterised by globose to subglobose or pyriform pycnidia, solitary terminal phialides conidiophores, ampulliform, lageniform or subcylindrical phialides, oblong, cylindrical or allantoid conidia, sometimes ellipsoid and 1-celled (Jaklitsch and Voglmayr 2016; Lu et al. 2022). In this study, we introduced one new species isolated from rubber tree, based on morphology and phylogeny.

#### ﻿ Nigrograna lincangensis

Taxon classificationFungiDyfrolomycetalesPleurotremataceae

R.F. Xu & Tibpromma sp. nov.

1AB76FA4-DD2F-5A36-8D5D-3A6BF4295FC0

Index Fungorum number: IF901420

Facesoffungi number: FoF15196

Fig. 4

##### Etymology.

The name refers to the location “Lincang, Yunnan, China”, where the holotype was collected.

##### Holotype.

ZHKU 23–0104.

##### Description.

***Saprobic*** on a dead branch of *Heveabrasiliensis*. ***Sexual morph***: Ascomata 285–360 μm high, 230–307 μm diam. (x‾ = 337 × 272 μm, n = 5), immersed, under the clypeus, sometimes inconspicuous on host surface and small bumps can be seen, solitary, dark brown, globose or ellipsoid, with papilla. Ostioles 117–217 × 68–124 μm (x‾ = 152 × 99 μm, n = 10), central, brown, papillate. Peridium 16–45 μm wide, comprising several layers with dark-brown to dark cells of textura angularis. Hamathecium comprises 1.5–3 μm wide, unbranched, septate, hyaline, pseudoparaphyses. Asci 45–70 × 9–12 μm (x‾ = 57 × 10 μm, n = 10), 8-spored, bitunicate, pedicellate, club shape, cylindrical to clavate, straight or slightly curved, apically rounded, thick-walled. Ascospores 10–15 × 4–6 μm (x‾ = 13 × 4.8 μm, n = 30), 1–2-seriate, initially 1-septate, becoming 3-septate at the maturity, fusoid to narrowly ellipsoid, upper part or second cell slightly wider and tapering towards narrow ends, constricted at the septa, hyaline to yellow-brown to brown with age, guttulate, think-walled. ***Asexual morph***: Undetermined.

##### Culture characteristics.

Spores germinated within 12 hours, colonies grow on PDA at 28 °C, circular, floppy, entire edge, raised, grey to taupe, reverse dark brown.

##### Material examined.

China, Yunnan Province, Lincang, on a dead branch of *Heveabrasiliensis*, 28 July 2022, Rui-Fang Xu, LCR06, (ZHKU 23–0104, holotype); ex-type ZHKUCC 23–0798, ZHKUCC 23–0799.

##### GenBank numbers.

ZHKUCC 23–0798 = ITS: OR853099, LSU: OR922323, SSU: OR941079, *tef*1-α: OR966282, *rpb*2: OR966280; ZHKUCC 23–0799 = ITS: OR853100, LSU: OR922324, SSU: OR941080, *tef1-α*: OR966283, *rpb*2: OR966281.

##### Notes.

**Figure 3.**
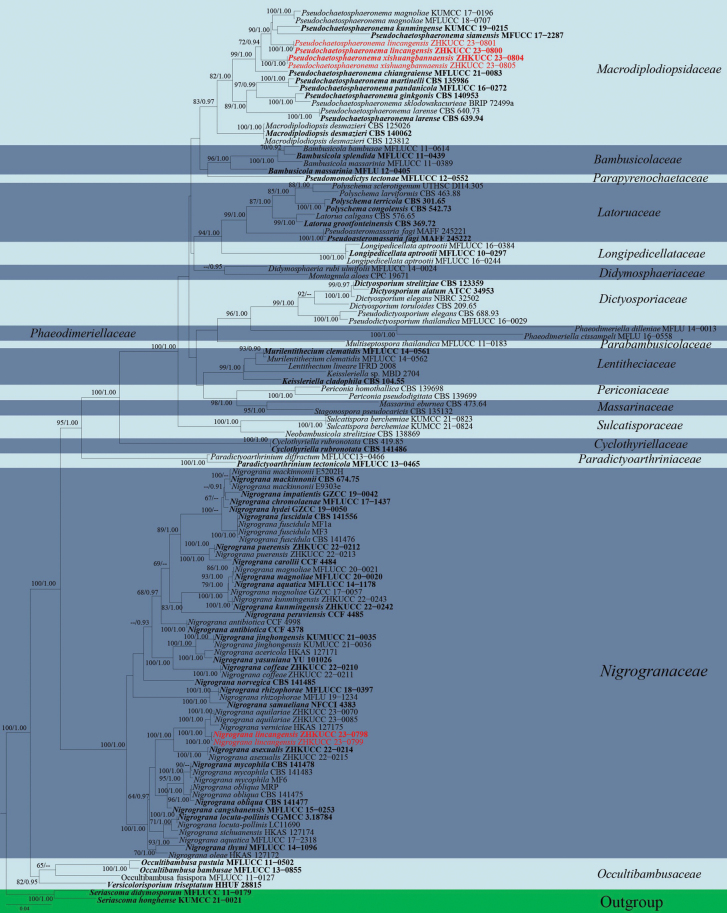
Phylogram generated from Maximum Likelihood analysis based on combined LSU, ITS, SSU, *tef*1-α and *rpb*2 sequence data of 119 taxa, which comprised 4399 base pairs (LSU = 908 bp, ITS = 512 bp, SSU = 1000 bp, *tef*1-α = 925 bp, *rpb*2 = 1054 bp). The best scoring RAxML tree with a final likelihood value of -38918.764563 is presented. The matrix had 2023 distinct alignment patterns, with 39.00% of undetermined characters or gaps. Estimated base frequencies were as follows: A = 0.245191, C = 0.247520, G = 0.268228, T = 0.239061; substitution rates: AC = 1.533778, AG = 3.877174, AT = 1.672983, CG = 1.254032, CT = 8.838860, GT = 1.000000; gamma distribution shape parameter α = 0.208600. Bootstrap support values for ML equal to or greater than 60% and Bayesian Inference analysis values equal to or greater than 0.90 PP are labelled at each node. The tree is rooted with *Seriascomadidymospora* (MFLUCC 11–0179) and *S.didymospora* (MFLUCC 11–0194). Related sequences were obtained from De Silva et al. (2022), Lu et al. (2022) and Li et al. (2023). The new isolates are indicated in red and the ex-type strains are in bold.

In the phylogenetic analyses, *Nigrogranalincangensis* (ZHKUCC 23–0798) forms a closely-related clade to *N.asexualis* (ZHKUCC 22–0214), *N.aquilariae* (ZHKUCC 23–0070) and *N.verniciae* with 100% ML and 1.00 PP support (Fig. 3). However, we could not compare the morphological characteristics of *N.lincangensis* and *N.asexualis*, because *N.lincangensis* was described only from its sexual morph in nature, while *N.asexualis* was described by its asexual morph in nature from coffee in China. A comparison of the ITS region of *N.lincangensis* and *N.asexualis* revealed 16 base pair differences (3.46%) across 462 nucleotides, 40 base pair differences (4.21%) across 949 nucleotides in *tef*1-α gene, 124 base pair differences (12%) across 1033 nucleotides in *rpb*2 gene. *Nigrogranaaquilariae* and *N.verniciae* have very similar morphological characteristics, but they can be differentiated by having wider ascomata (285–360 μm vs. 180–270 μm), larger asci (45–70 × 9–12 μm vs. 49–57 × 7–9 μm) and larger ascospores (10–15 × 4–6 μm vs. 10–13 × 3.5–4.5 µm) in *N.lincangensis* (Du et al. 2024); while *N.verniciae* has larger ascomata (340–360 × 350–370 μm vs. 85–360 μm × 230–307 μm) and asci with knob-like to furcate pedicels (Li et al. 2023).

*Nigrogranalincangensis* has similar ascomata, asci and ascospore characteristics similar to other *Nigrograna* species (Jaklitsch and Voglmayr 2016; Hyde et al. 2017; Tibpromma et al. 2017; Dayarathne et al. 2020; Mapook et al. 2020; Lu et al. 2022). However, *N.lincangensis* differs from *N.cangshanensis* by having larger ascomata (285–360 × 230–307 μm vs. 120–135 × 135–155 μm) (Tibpromma et al. 2017). *Nigrogranachromolaenae* can be distinguished from *N.lincangensis* in having smaller ascomata (160–280 × 115–130 μm vs. 285–360 × 230–307 μm), smaller asci (40–55 × 7–10 μm vs. 45–70 × 9–12 μm), and greyish-brown to dark brown ascospores (Mapook et al. 2020). *Nigrogranacoffeae* differs from *N.cangshanensis* by having smaller ascomata (90–140 × 140–200 μm vs. 285–360 × 230–307 μm), 1-septate ascospores (Lu et al. 2022). *Nigrogrananovergica* differs from *N.lincangensis* as it occurs on pseudostromata from the host of *Diaporthe* sp. (Jaklitsch and Voglmayr 2016). *Nigrogranamycophila* and *N.obliqua* are distinct from *N.lincangensis* by having dark brown ascospores (Jaklitsch and Voglmayr 2016). *Nigrogranapuerensis* differs from *N.lincangensis* by having acute apical and basal cells and the apical cells are wider than the basal cells (Lu et al. 2022). *Nigrogranasamueliana* differs from *N.lincangensis* by the absence of ostiole (Dayarathne et al. 2020). *Nigrogranathymi* can be easily distinguished from *N.lincangensis* in having 4–5 septate (Hyde et al. 2017). Therefore, *N.lincangensis* is described here as a new species, based on phylogeny and morphology.

**Figure 4.**
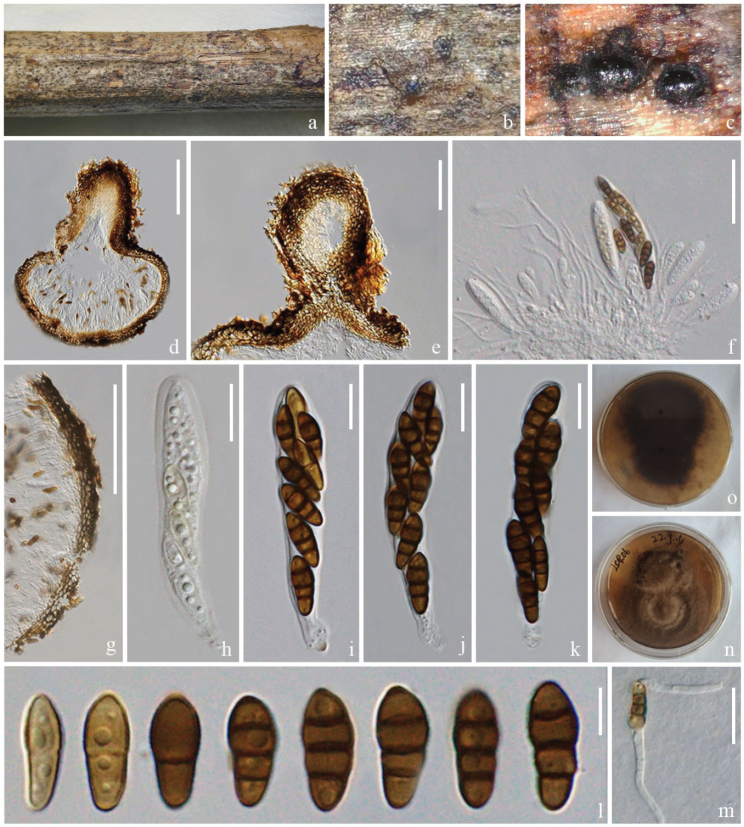
*Nigrogranalincangensis* (ZHKU 23–0104, **holotype**) **a–c** appearance of ascomata on the host surface **d** vertical section of an ascoma **e** vertical section of ostiole **f** hamathecium and asci **g** section of peridium **h–k** asci **l** ascospores **m** a germinated ascospore **n, o** colonies on PDA (n-front and o-reverse views). Scale bars: 100 µm (**d**); 50 µm (**e**); 30 µm (**f**); 200 µm (**g**); 10 µm (**h–k**); 5 µm (**l**); 20 µm (**m**).

#### ﻿ Macrodiplodiopsidaceae

Taxon classificationFungiDyfrolomycetalesPleurotremataceae

Voglmayr, Jaklitsch & Crous

E7146F67-0544-5AE8-A461-36B87DF91A50

##### Notes.

Macrodiplodiopsidaceae was introduced by Crous et al. (2015) with *Macrodiplodiopsis* as the type genus. There are two genera viz. *Macrodiplodiopsis* and *Pseudochaetosphaeronema* in this family (Wijayawardene et al. 2022).

#### ﻿ Pseudochaetosphaeronema

Taxon classificationFungiDyfrolomycetalesPleurotremataceae

Punith.

119D0C27-97DF-57A1-83CF-4CE6FCF4EF22

##### Notes.

*Pseudochaetosphaeronema* was introduced by Punithalingam (1979), with *P.larense* as the type species. The members of this genus have been reported as human pathogens, endophytes and saprobes (Boonmee et al. 2021). Nine epithets are listed in Index Fungorum (2024), i.e. one sexual *P.chiangraiense* and eight asexual species viz. *P.ginkgonis*, *P.kunmingense*, *P.larense*, *P.magnoliae*, *P.martinelli*, *P.pandanicola*, *P.siamense* and *P.sklodowskacurieae*. The asexual morph of *Pseudochaetosphaeronema* is characterised by globose, conidiomata, monophialidic, cylindrical conidiogenous cells and hyaline, subglobose to oval, aseptate conidia (De Silva et al. 2022). The sexual morph is characterised by immersed, uni-loculate ascomata, peridium with the cells of textura angularis, unbranched, septate pseudoparaphyses, 8-spored, bitunicate, fissitunicate, short distinct pedicel asci with rounded end and fusiform, 1-septate, guttulate ascospores with pointed ends (Boonmee et al. 2021).

#### ﻿ Pseudochaetosphaeronema lincangensis

Taxon classificationFungiDyfrolomycetalesPleurotremataceae

R.F. Xu & Tibpromma sp. nov.

AA520AD2-DE95-5072-8D54-6E125383EF49

Index Fungorum number: IF901421

Facesoffungi number: FoF15197

Fig. 5

##### Etymology.

The name refers to the location “Lincang, Yunnan, China”, where the holotype was collected.

##### Holotype.

ZHKU 23–0105.

##### Description.

***Saprobic*** on a dead branch of *Heveabrasiliensis*. ***Sexual morph***: Ascomata 140–245 μm high, 255–290 μm diam., (x‾ = 190 × 267 μm, n = 5), immersed, visible as dark-brown dots on the host surface, solitary, uni-loculate, ampulliform, without ostiole. Peridium 18–50 μm wide, several layers, comprising dark-brown to pale-brown cells of textura angularis. Hamathecium comprises 2–3 μm wide, numerous, hyaline, unbranched, pseudoparaphyses. Asci 90–145 × 15–30 μm (x‾ = 112 × 22 μm, n = 15), 8-spored, bitunicate, cylindrical to clavate, apically rounded, short pedicelate, with a small ocular chamber, thick-walled. Ascospores 25–40 × 8–15 μm (x‾ = 30 × 11 μm, n = 35), overlapping, 2-seriate, fusiform, 1-septum in the middle of cell, widest at the centre and tapering towards narrow ends, constricted at the septum, hyaline, guttulate, with ellipsoid mucilaginous sheath, thick and smooth-walled. ***Asexual morph***: Undetermined.

**Figure 5.**
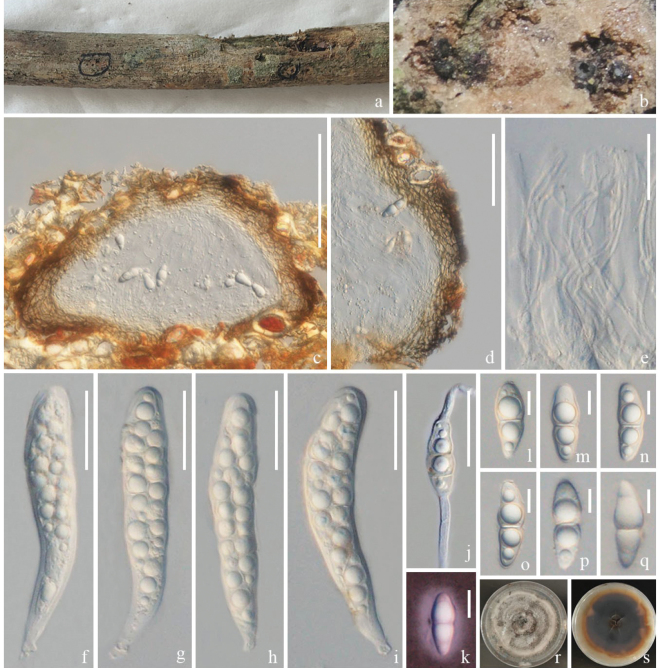
*Pseudochaetosphaeronemalincangensis* (ZHKU 23–0105, **holotype**) **a, b** appearance of ascomata on host substrate **c** vertical section of an ascoma **d** section of peridium **e** pseudoparaphyses **f–i** asci **j** a germinated ascospore **l–q** ascospores **k** ascospore stained with Indian ink **r, s** colonies on PDA (r-front and s-reverse views). Scale bars: 100 µm (**c**); 50 µm (**d**); 30 µm (**e–j**); 200 µm (**g**); 10 µm (**k, l–q**).

##### Culture characteristics.

culture on PDA, colonies slow growing on 28 °C, low convex, entire, smooth, edge is off-white from above, dark brown, edge is orange on reverse side.

##### Material examined.

China, Yunnan Province, Lincang on a dead branch of *Heveabrasiliensis*, 28 July 2022, Rui-Fang Xu, LCR07 (ZHKU 23–0105, holotype); ex-type ZHKUCC 23–0800, ZHKUCC 23–0801.

##### GenBank numbers.

ZHKUCC 23–0800 = ITS: OR853095, LSU: OR922336, SSU: OR922342, *tef*1-α: OR966290; ZHKUCC 23–0801 = ITS: OR853096, LSU: OR922337, SSU: OR922343, *tef*1-α: OR966291.

##### Notes.

In the phylogenetic analyses, *Pseudochaetosphaeronemalincangensis* clusters distinctly, sister to *P.kunmingense*, *P.magnoliae* and *P.siamensis* with 90% MP, 1.00 PP support (Fig. 3). The base pair differences in ITS, LSU, SSU and *tef*1-α sequences of our new species are compared with *P.kunmingense*, *P.magnoliae* and *P.siamensis* (Table 3). However, we could not compare the morphological characteristics of the species above, as they were described, based on asexual morphs. Therefore, based on morphology and phylogeny, we introduce *Pseudochaetosphaeronemalincangensis* as a new species.

**Table 3.** Nucleotide differences in the ITS, LSU, SSU and *tef*1-α of *P.lincangensis* (ZHKUCC 23–0800) compared with *P.kunmingense*, *P.magnoliae* and *P.siamensis*.

Strains	ITS	LSU	SSU	*tef*1-α
*P.kunmingense* (KUMCC 19–0215)	30/506 (5.93%)	10/855 (1.16%)	4/1012 (0.39%)	38/893 (4.26%)
*P.magnoliae* (KUMCC 17–0196)	51/539 (9.46%)	19/854 (2.22%)	8/939 (0.85%)	32/899 (3.56%)
*P.siamensis* (MFUCC 17–2287)	43/480 (8.96%)	11/848 (1.29%)	1/1005 (0.09%)	98/645 (15.19%)

#### ﻿ Pseudochaetosphaeronema xishuangbannaensis

Taxon classificationFungiDyfrolomycetalesPleurotremataceae

R.F. Xu & Tibpromma sp. nov.

EAECAD44-38E7-5AAC-9586-CA3E531C6A3B

Index Fungorum number: IF901422

Facesoffungi number: FoF15198

Fig. 6

##### Etymology.

The name refers to the location “Xishuangbanna, Yunnan, China”, where the holotype was collected.

##### Holotype.

ZHKU 23–0107.

##### Description.

***Saprobic*** on a dead branch of *Heveabrasiliensis*. ***Sexual morph***: Ascomata 270–410 μm high, 370–480 μm diam., (x‾ = 350 × 420 μm, n = 5), solitary, scattered, immersed, globose to subglobose, uni-loculate, black. Peridium 40–90 μm wide, thin-walled, composed of several layers of small, brown to pale brown cells of textura intricata. Hamathecium comprises 2–3 μm wide, numerous, dense, filiform, unbranched, hyaline, cellular pseudoparaphyses. Asci 130–180 × 25–35 μm (x‾ = 155 × 32 μm, n = 20), 8-spored, bitunicate, obovoid, short distinct pedicel with conical end, apex rounded with a minute ocular chamber. Ascospores 30–50 × 10–20 μm (x‾ = 42 × 13 μm, n = 30), hyaline, fusiform, with pointed ends, 3–5-septate, larger upper third cell, constricted at the septa, guttulate, thick-walled, with mucilaginous sheath, the sheath constricted at the middle. ***Asexual morph***: Undetermined.

**Figure 6.**
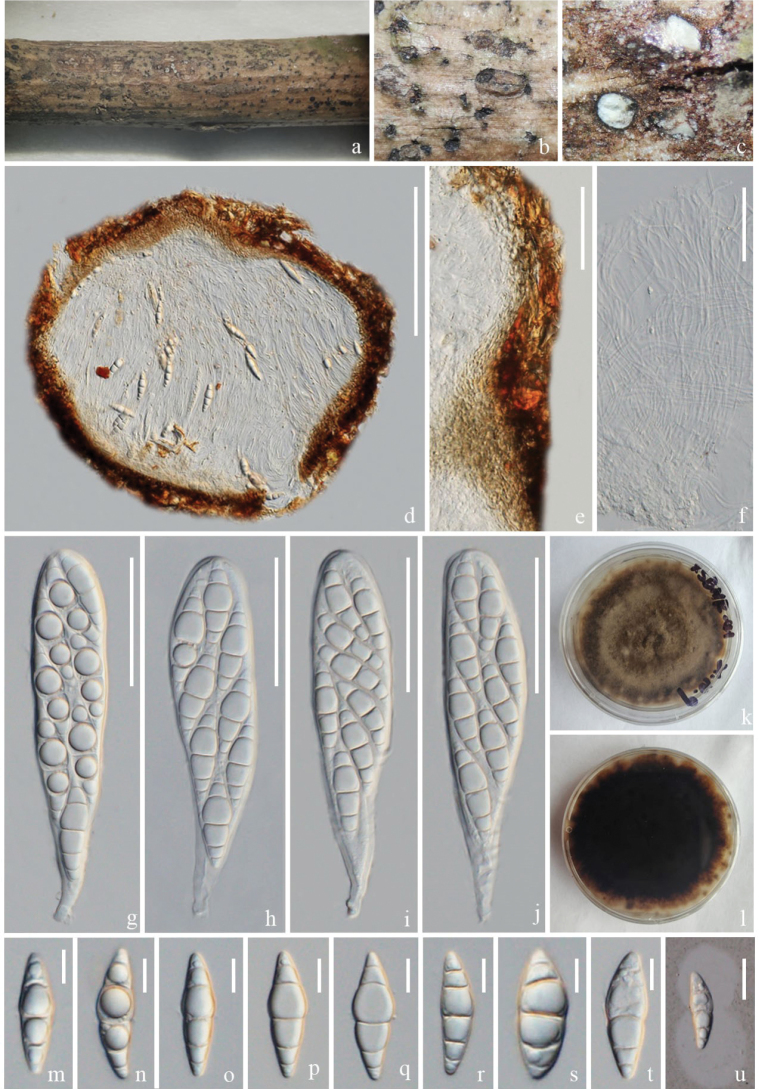
*Pseudochaetosphaeronemaxishuangbannaensis* (ZHKU 23–0107, **holotype**) **a–c** appearance of ascomata on host substrate **d** section of an ascoma **e** peridium **f** pesudoparaphyses **g–j** asci **m–t** ascospores **u** ascospore stained with Indian ink **k, l** colonies on PDA (k-front and l-reverse view). Scale bars: 200 µm **(d)**; 100 µm **(e)**; 50 µm **(f–j)**; 10 µm **(m–t)**; 20 µm **(u)**.

##### Culture characteristics.

Colony on PDA, colonies slow growing on 28 °C, umbonate, filiform, smooth, edges brown, from above, brown, dark brown on reverse side.

##### Material examined.

China, Yunnan Province, Xishuangbanna on a dead branch of *Heveabrasiliensis*, 12 September, 2021, Rui-Fang Xu, XSBNR–41 (ZHKU 23–0107, holotype); ex-type ZHKUCC 23–0804, ZHKUCC 23–0805.

##### GenBank numbers.

ZHKUCC 23–0804 = ITS: OR853097, LSU: OR922338, SSU: OR922344, *tef*1-α: OR966286; ZHKUCC 23–0805 = ITS: OR853098, LSU: OR922339, SSU: OR922345, *tef*1-α: OR966287.

##### Notes.

In the phylogenetic analyses, *Pseudochaetosphaeronemaxishuangbannaensis* clusters with *P.lincangensis* with 99% ML and 1.00 PP support (Fig. 3). Morphologically, *P.xishuangbannaensis* differs from *P.lincangensis* in having longer asci (130–180 μm vs. 90–145 μm), 3–5-septate ascospores with sheath constricted at the central septum and brown to dark brown colonies, while *P.lincangensis* has ascospores with a normal sheath in a circle, 1-septate ascospores with obtuse ends and colonies off-white from the forward edge, orange in reverse. *Pseudochaetosphaeronemaxishuangbannaensis* shares similar morphologies with *P.chiangraiense*, but can be differentiated by having the peridium with the cells of *textura intricate*, larger ascomata (270–410 × 370–480 μm vs. 190–255 × 190–200 μm), longer asci (130–180 μm vs. 50–110 μm), larger (30–50 × 10–20 μm vs. 20–45 × 15–30 μm) and 3–5 septate ascospores with a sheath constricted at the central septum and brown to dark brown colonies. *Pseudochaetosphaeronemachiangraiense* has textura angularis peridium, ascospores surrounded by a normal sheath in a circle, 1-septum, obtuse ends, from above, greenish-grey in the middle and pale brown at the margin, yellowish-brown on the reverse side (Boonmee et al. 2021). In addition, *P.xishuangbannaensis* formed a different lineage with *P.chiangraiense* (Fig. 3). Therefore, *P.xishuangbannaensis* is described as a new species, based on phylogenetic analyses and morphological comparison.

## ﻿Discussion

Global fungal diversity is astounding. Although around 155,000 fungal species have been described, up to 19 million have yet to be described (Hyde 2022; Phukhamsakda et al. 2022). Fungi have been classified into five different phyla: Chytridiomycota, Zygomycota, Glomeromycota, Ascomycota and Basidiomycota (Aguilar-Marcelino et al. 2020; Wijayawardene et al. 2022). Fungi play an important role in litter decomposition by breaking down lignin and other refractory components in the litter, thereby affecting the decomposition of terrestrial ecosystems, especially by activities of Basidiomycota and Ascomycota (Osono and Takeda 2002; Bucher et al. 2004; Phukhamsakda et al. 2022). Discovering more saprophytic fungi associated with rubber will enrich our knowledge on saprobic fungi and their functions as litter degraders. Microfungi from warm climates have a more significant decomposition capacity than from cool climates (Osono et al. 2011). Previous studies have reported that Ascomycota, Basidiomycota and Oomycota are abundant on Pará rubber leaf and branch litter (Monkai et al. 2017; Meeboon and Takamatsu 2020; Senwanna et al. 2021). Nizamani et al. (2023) provided a checklist comprising 788 species and 179 taxa identified at the genus level from 57 countries. The taxa listed in the checklist belong to 515 genera, 180 families and 68 orders and more than half of these taxa were isolated from leaf and branch litter.

In Southeast Asia, Pará rubber plantations have been expanding rapidly since the 20^th^ century and, currently, supply over 90% of the world’s natural rubber (Fox and Vogler 2005; Mann 2009; Ziegler et al. 2009). More than one million hectares of lands in Cambodia, Laos, Myanmar, South China, Thailand and Vietnam have been converted into Pará rubber plantations (Li and Fox 2012). In 1904, China planted rubber for the first time in Yingjiang, Dehong, in Yunnan Province (Chapman 1991). Pará rubber is widely cultivated in the Hainan, Guangdong, Guangxi, Fujian and Yunnan Provinces in China as an economically important plant (Wang et al. 2015).

Pará rubber is vulnerable to many pests and diseases, but it is still a mystery why only a few fungal species have been found on rubber (Senwanna et al. 2021). Pará rubber tree was introduced to China from Malaysia, presumably by seed and endemic fungi are unlikely to follow; therefore, new fungi colonise Pará rubber through host-shifting or host-jumping (Roy 2001; Senwanna et al. 2019, 2021). Fungi associated with Pará rubber are found in different life modes such as saprobic, endophytic and pathogenic (Gazis and Chaverri 2010; Monkai et al. 2017; Senwanna et al. 2021). On Pará rubber, Dothideomycetes predominate amongst ascomycetes (Senwanna et al. 2021) and four species described in our study also belong to Dothideomycetes.

Fungal pathogens and endophytes were also isolated from the Pará rubber trees. Additionally, studies have been conducted to analyse the richness and diversity of endophytic fungi in different tissues of *Heveabrasiliensis* (Martin et al. 2015; Rojas-Jimenez et al. 2016; Araújo et al. 2020). Pathogens cause potential disease threats to *Heveabrasiliensis*; for example, *Corynesporacassiicola* causes *Corynespora* leaf fall disease (Jayasinghe and Fernando 1998), *Microcyclusulei* causes South American leaf blight (Júnior et al. 2014), the basidiomycete genera *Phellinus*, *Rigidoporus* and *Ganoderma* cause stem- and root-rots (Mohammed et al. 2014). In addition, the estimated richness of endophytic fungi does not significantly differ amongst the leaves, stems and roots; and the fungal diversity is higher in the stems and roots compared to the leaves (Martin et al. 2015; Araújo et al. 2020). Mahendran et al. (2021) revealed that *Aspergillusterreus* has a good inhibitory potential against *Rigidoporusmicroporus* and *Corynesporacassiicola* and has potential for biological control. Therefore, it is important to understand the fungi associated with Pará rubber trees to manage and prevent rubber tree diseases.

Only a few reports are available for the saprobic fungi on *Heveabrasiliensis* in China and many taxa lack molecular data (Seephueak et al. 2010, 2011; Senwanna et al. 2021). Therefore, a revised taxonomic approach with multi-gene phylogenetic analyses is necessary to understand the fungal diversity associated with Pará rubber. In this study, we introduce four new saprobic fungi from branches and twigs of rubber trees, based on morphology and molecular phylogenetic analyses. This enriches the fungal diversity in Pará rubber and provides information for host jumping.

## Supplementary Material

XML Treatment for Pleurotremataceae

XML Treatment for Melomastia

XML Treatment for Melomastia puerensis

XML Treatment for Nigrogranaceae

XML Treatment for Nigrograna

XML Treatment for Nigrograna lincangensis

XML Treatment for Macrodiplodiopsidaceae

XML Treatment for Pseudochaetosphaeronema

XML Treatment for Pseudochaetosphaeronema lincangensis

XML Treatment for Pseudochaetosphaeronema xishuangbannaensis

## ﻿Additional information

### Author ORCIDs

Rui-Fang Xu https://orcid.org/0000-0003-1207-8254

Samantha C. Karunarathna https://orcid.org/0000-0001-7080-0781

Chayanard Phukhamsakda https://orcid.org/0000-0002-1033-937X

Dong-Qin Dai https://orcid.org/0000-0001-8935-8807

Abdallah M. Elgorban https://orcid.org/0000-0003-3664-7853

Nakarin Suwannarach https://orcid.org/0000-0002-2653-1913

Jaturong Kumla https://orcid.org/0000-0002-3673-6541

Xiao-Yan Wang https://orcid.org/0009-0009-6430-3637

Saowaluck Tibpromma https://orcid.org/0000-0002-4706-6547

### Data availability

All of the data that support the findings of this study are available in the main text.
